# Role of interleukin-16 in human diseases: a novel potential therapeutic target

**DOI:** 10.3389/fimmu.2025.1524026

**Published:** 2025-06-02

**Authors:** Timothy B. Niewold, Julia S. Lehman, Iva Gunnarsson, Alexander Meves, Vilija Oke

**Affiliations:** ^1^ Barbara Volcker Center for Women and Rheumatic Disease, Hospital for Special Surgery, New York, NY, United States; ^2^ Department of Dermatology, Mayo Clinic, Rochester, MN, United States; ^3^ Rheumatology Unit, Department of Meidicine, Karolinska Institutet, Stockholm, Sweden; ^4^ Rheumatology, Theme Inflammation and Aging, Karolinska University Hospital, Stockholm, Sweden; ^5^ Academic Specialist Center, Stockholm Region, Stockholm, Sweden

**Keywords:** cytokine - immunological terms, interleukin-16, inflammation, infection, cancer, tumor

## Abstract

Interleukin (IL)-16 is expressed mostly by the cells of the human immune system. Upon cell activation IL-16 is cleaved, forming two functional proteins, one regulating cell cycle and the other acting as chemoattractant for the cells carrying CD4 or CD9. Increased levels of IL-16 are found in the circulation and at the sites of inflammation, infection and cancer. Polymorphisms in the IL-16 gene have been coupled to several of these conditions and high IL-16 has been suggested as a disease biomarker. Using unbiased proteomic approach we and others independently identified IL-16 as a biomarker of severe lupus nephritis, and top-expressed cytokine in skin lesions of lupus erythematosus. Recently, an unbiased investigation identified IL-16 as a top candidate for novel drug target. Blockade of IL-16 showed positive therapeutic effects in several animal models of human disease with low rate of side effects. Importantly, it has been recently demonstrated that IL-16 can be released during pyroptosis, a proinflammatory cell death pathway. This finding disclosed a novel role of IL-16 as a mediator of response to the proinflammatory cell death and may explain why IL-16 is detected at the sites of inflammation, infection or cancer. In this review we cover the knowledge on the biology of IL-16 and its importance in human diseases. We aim that this manuscript will be informative and prove benefits of possible therapeutic blockade of IL-16.

## 1 Biology of IL-16

### 1.1 Characteristics of IL-16 protein

**Figure 1:**
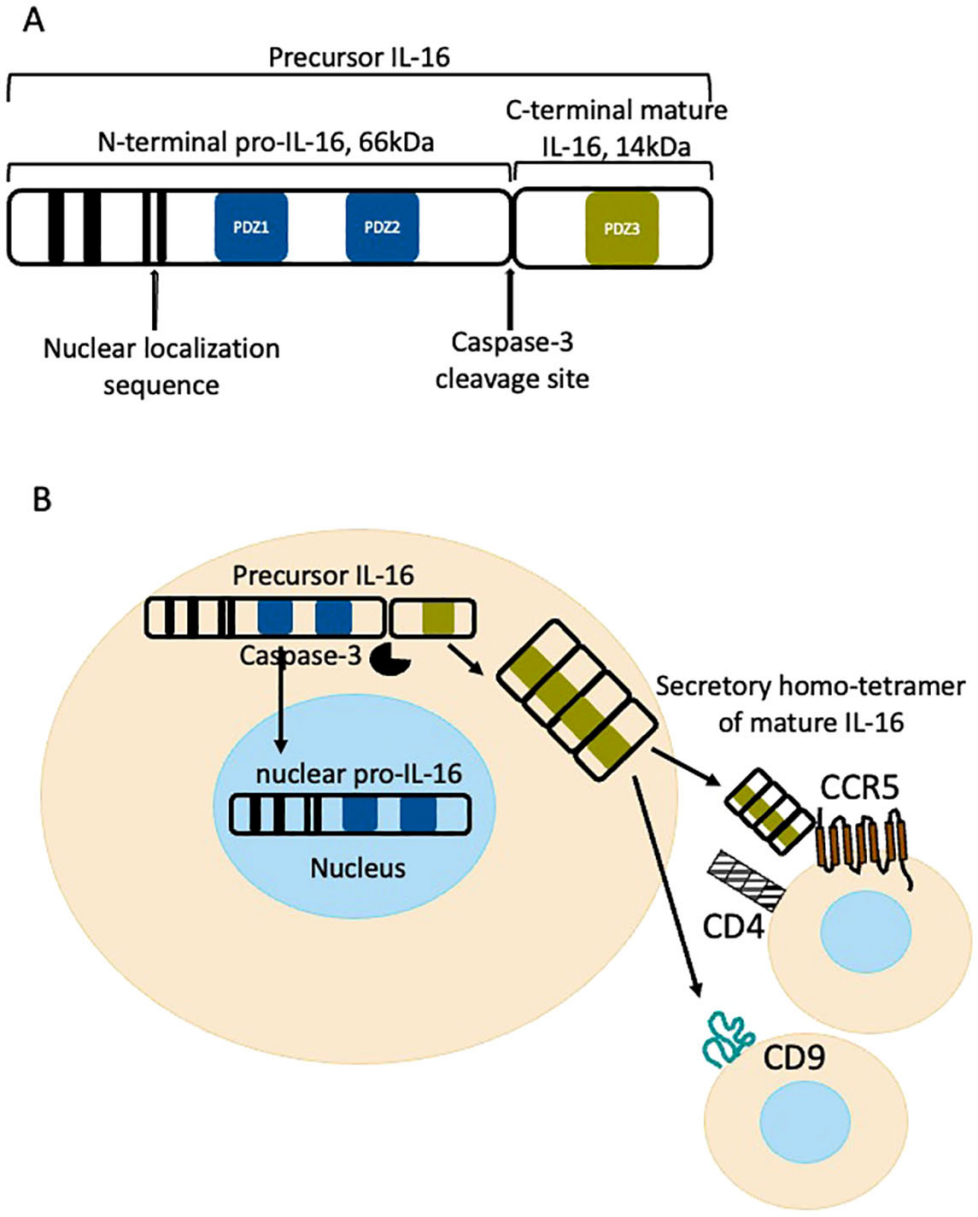
**(A)** Structure of IL-16 protein. **(B)** Processing of IL-16 within a cell. The cytoplasmic IL-16 is cleaved by active caspase-3 and mature C terminal IL-16 forms homo-tetramers than can be secreted and ligate its receptors CD4, CD9 or CCR5. N terminal pro-IL-16 enters nucleus and regulate cell cycle.

**Figure 2:**
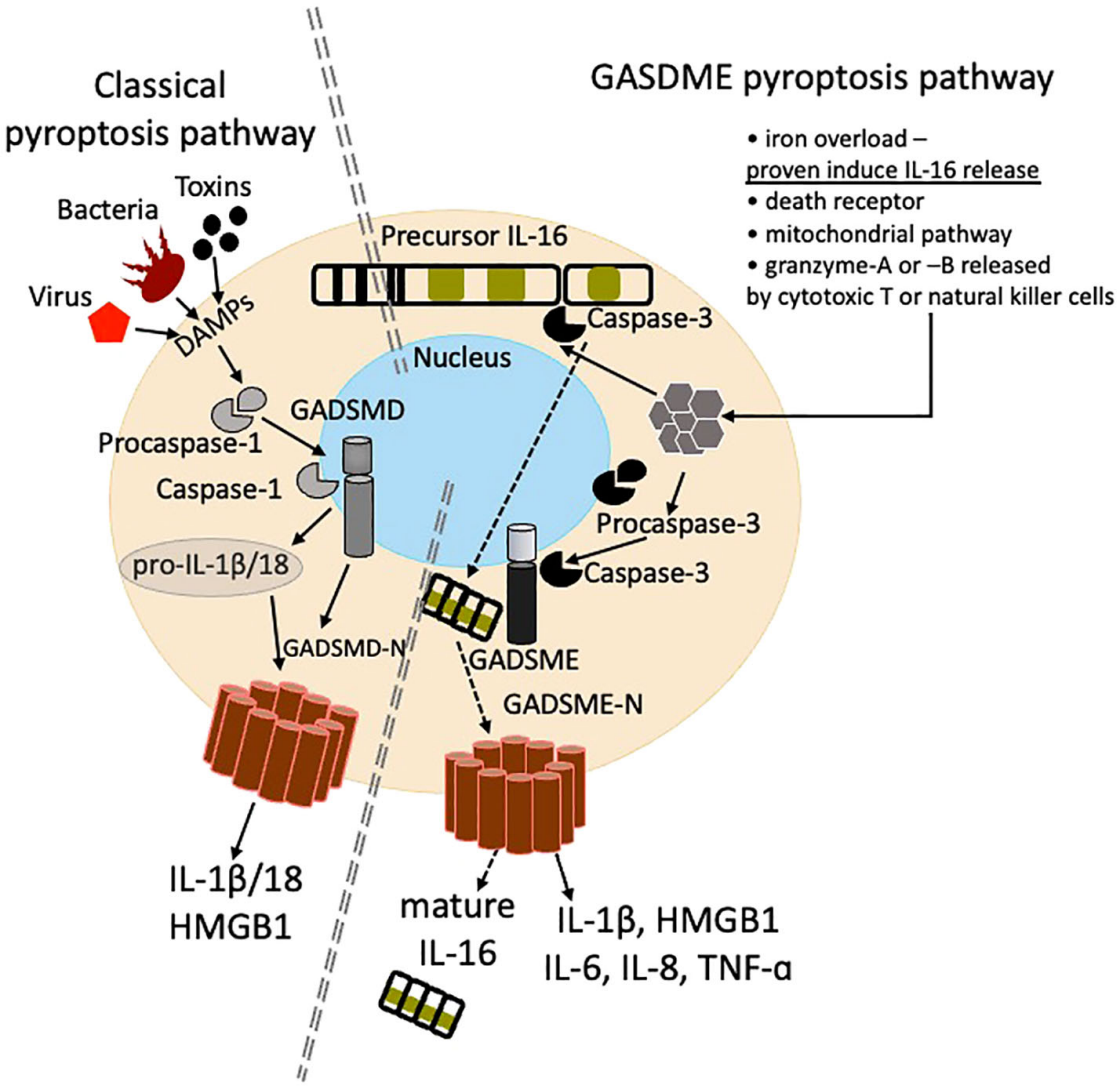
Schematic illustration of classical pyroptosis pathway (left) and a relatively novel gasdermin-E (GSDME) pyroptosis pathway (right) resulting in IL-16 cleavage and release. The classical pyroptosis pathway is induced by recognition of danger associated molecular patterns (DAMPS), activation of inflammasome, cleavage of procaspase-1 to caspase -1, subsequent cleavage of GASDM-E and pro-IL-1b/18, multimerization of GASDME-N terminal products and a cell pore formation and release of proinflammatory mediators including IL-1b/18. The GASDME pathway can be activated by several stimuli including granzyme A and B, iron overload, extrinsic and intrinsic death receptors, and mitochondrial damage.

The human IL-16 gene is encoded on chromosome 15q26.3, is 153 kilobase pair (kbp) long and consists of seven exons and six introns (1). IL-16 protein is highly conserved among species. Two translated IL-16 proteins have been found in humans: one is restricted to the central nervous system, entitled neuronal NIL-16 (130 kilo daltons (kDa) or 141kD according different sources) (2). The 80 kDa IL-16 is found in many other organ tissues, particularly lymphoid, and is generated as a 631-amino-acid precursor IL-16 molecule. After cell stimulation caspase-3 (casp-3) may cleave IL-16 at residue asparagine^253^ into 66kDa N terminal also called pro-IL-16 and 14kDa C terminal mature IL-16 (**Figure 1A**) (3–5). IL-16 is highly conserved, up to 96-98%, across the species close to humans such as Rhesus, Macaques and monkeys (3, 6).It is reported that functional activity of the secreted mature IL-16 is localized at the hydrophylic region of the carboxyterminal, and before secretion 14kDA chains form 56kDa homotetramers (**Figure 1B**) (6). The N terminus of IL-16 can be located in either the cytosol or the nucleus of the cells and may regulate cell growth (7, 8). Cleaved or mature IL-16 is supposed to be secreted; however, certain cell types (i.e. cluster differentiation (CD) 8+ T cells) may contain cytoplasmic stores of mature, bioactive IL-16 which can be released upon stimulation, saving time from new synthesis (9, 10). Secretion of bioactive IL-16 is regulated by mitogen-activated protein (MAP) kinase and has been postulated to be likened to secretion of IL-1β following cleavage by caspase-1 (11). IL-16 mRNA is regulated in calcineurin dependent manner (12). Casp-3 activation and IL-16 cleavage is not necessarily associated with the induction of apoptosis, but recently release of IL-16 has been described in endometriosis via Gasdermin (GASDM) E pyroptotic pathway (**Figure 2**) (9, 13).

### 1.2 Cellular expression of IL-16 in healthy tissues and their cells

Physiologically IL-16 is expressed by cells of lymphoid organs: bone marrow, spleen, lymph nodes, thymus, tonsils and appendix, while tissues of the lower gastrointestinal tract express IL-16, but at a lower level. The majority of other tissues express mRNA for IL-16, and presumably can translate and secrete IL-16 under certain conditions. Among other tissue cells IL-16 has been found in cutaneous, cardial, renal fibroblasts and bronchial epithelial cells (14–16).

The majority of the immune cell types can express IL-16 protein. The CD4+ and CD8 positive T cells are the best studied expressors of IL-16, but also neutrophils, B-cells, eosinophils, dendritic cells, mast cells, macrophages and monocytes, natural killer cells all are found to express IL-16 (9, 17–20). Microglial cells and astrocytes are also found to express IL-16, while neurons did not show IL-16 expression (21).

It seems that IL-16 is a rapid response cytokine in certain cell types, for example in CD8+ T cells, eosinophils and also mast cells, where circulating cells carry an already pre-synthesized protein and are ready to release it immediately after stimulation with vasoactive amines, such as serotonin or histamine (22).

Reports indicate that same types of cells express and secrete IL-16 in various categories of human diseases including infectious, inflammatory diseases and cancers. The overall data point that levels of circulating IL-16 are increased in the investigated diseases. Knowledge on what cell types are the producers and responders in respective conditions are limited and is covered in more details below. Data indicates that mechanisms which tune IL-16 are of importance and modulation might have therapeutic effect.

### 1.3 Receptors of the IL-16

Several receptors for IL-16 have been identified: CD4, CD9 and CCR5 (3, 23, 24). CD4 is regarded as the major receptor as it is expressed by a range of hematopoietic and immune cells, as well on neuronal cells (3). The main ligand of IL-16 is D4 domain of CD4 (3). Many types of lymphoid cells including T cells, eosinophils, dendritic cells, B cells, as well as mast cells, macrophages and monocytes respond to IL-16, but it is not completely clear what receptors IL-16 binds (15, 19, 24–28). In the spinal cord CD4 is expressed in microglia, located close to astrocytes and neurons (29). The best described cellular function of IL-16 is mediated by CD4 binding, which induces cell migration via chemoattraction. Upon binding to the D4 domain of CD4, IL-16 activates the CD4-associated src-related tyrosine kinase p56lck via autocatalysis (30). Protein kinase C subsequently translocate to the membrane, inducing downstream signaling pathways in the target cell, including a rise in intracellular calcium, inositol trisphosphate (IP) 3 and phosphoinositide 3-kinase (PI3K) activation (30, 31). Additionally, IL-16 has also been shown to activate signal transducer and activator of transcription (STAT) 6 following interaction with CD4, though downstream signaling pathways and subsequent cellular responses have not yet been identified (31). Co-ligation of C-C chemokine receptor type 5 (CCR5) enhances cell response to the IL-16 signaling, and importantly CCR5 is preferentially expressed on T helper (Th) 1 cells, what seems to enhance migratory IL-16 effects on these cells (24). Findings indicate that before secretion, IL-16 preferentially forms multimers (homodimers or homo-tetramers) to mediate its effects more efficiently (4). Multimerization of IL-16 facilitates crosslinking of CD4 and CCR5 on the target cells and enhances cell signaling (4). Several studies have shown that IL-16/CD4 ligation may result in desensitization for other chemokine receptors, such as CCR5-, C-X-C chemokine receptor type (CXCR) 3 and CXCR4-induced migration (32), impairing macrophage inflammatory protein-1 beta (MIP-1b) and stromal cell-derived factor 1 (SDF-1a) mediated T cell chemoattraction (23, 24, 32, 33). In the presence of IL-16, the chemokine ligands for receptors including C-C motif chemokine ligand 5 (CCL5 or RANTES), MIP-1a, MIP-1, and SDF-1a are unable to recruit T cells, which indicates that IL-16 may direct what cells should be recruited (9).

The cells of monocyte and epithelial origin may respond to IL-16 via CD9 receptor as demonstrated *in vitro* on primary cells, cell lines and also in mouse models (15, 25, 34, 35).

### 1.4 The physiologic importance of IL-16 in the regulation cell cycle and cell growth

Both the N-terminal pro-IL-16 and the secreted mature IL-16 may impact cell growth. Cleaved N-terminal or pro-IL-16 fragment translocate into the nucleus and has been suggested to serve as cell cycle regulator (10). Repeated studies confirmed that pro-IL-16 has a cell cycle regulator function in T cells: high nuclear expression of pro-IL-16 induces cell cycle arrest G0/G1, inhibit cell proliferation and is associated with increased levels of cyclin-dependent kinase inhibitor p27Kip1 (36). During T cell activation, mRNA of IL-16 is downregulated and less IL-16 is observed in cell nuclei, which may result in expression of S-phase kinase-associated protein 2 (Skp2), degradation of p27Kip1 and enable the cell to enter cell cycle (36).

It could render stimulated and pre-exposed T cells refractory to antigenic stimulation, promoting anergy (37).

## 2 The major cellular sources of IL-16 is the human immune system

All major types of immune cells express IL-16. Overview of data is presented in **Table 1**.

**Table 1:** Expression of IL-16 in different immune cell types.

Cell type	IL-16 inducing signal	Outcome	Ref.
CD4+ T cells CD8+ T cells	Antigens, Mitogens, Vasoactive amines	Cleavage by activated casp-3, secretion, chemotaxis of Th1 cells	(34)
B cells	ND	Chemotaxis, recruitment of T cells, dendritic cells	(38, 39)
Neutrophils	Secondary necrosis	Chemotaxis, recruitment of neutrophils	(40, 41)
Monocytes, macrophages	Spontaneous in room temperature and time after sample withdrawal Infection, interferon-γ and lipopolysaccharides	ND Increased phagocytosis	(42), (43)
Dendritic (DC) and Langerhans cells	IgE bound allergens	Cleavage by activated casp-1, secretion, attraction of DCs, T cells, eosinophils	(44)
Mast cells	ND	Chemotaxis, activation	(25)
NK cells	ND	ND	(38)
Eosinophils	ND	Migration, persistent activation of eosinophils	(45, 46)
Epithelial cells	ND	ND	(15, 34, 35)

Ref., references; ND, no data.

### 2.1 IL-16 and T cells

Human T cells are the most studied source and target of IL-16. T cell activation by antigens, mitogens or vasoactive amines (like histamine or serotonin) may result in activation of casp-3 and cleavage of mature IL-16, which can be secreted. This process was described in both CD4+ and CD8+ T cells. While casp-3 is constantly active in CD8+ cells, continuously produced and cleaved IL-16 is stored in secretory vesicles, activation of casp-3 in CD4+ T cells must be induced by a stimuli, and thereafter IL-16 can be cleaved (34). Interestingly, it may take different amount of time from cell stimulation to IL-16 secretion depending on what stimuli the CD8+ cell receives: for histamine and serotonin it takes several hours, while for antigens and mitogens it requires 12–24 hours. Importantly, addition of transcription and translation inhibitors may block IL-16 secretion from CD4+, but not from CD8+ cells (reviewed in (4)). *In vitro*, IL-16 treatment of human peripheral CD4+T lymphocytes induces upregulation of IL-2Ra (CD25) and IL-2Rb within 24 h, enhancing IL-2 mediated T cell proliferation (9, 47). Binding of bioactive IL-16 to CD4 preferentially induces chemotaxis of CD4+Th1 type T cells, rather than Th2 type (24). In the presence of CCR5 the response to IL-16 is enhanced, while IL-16 could not bind to CCR5 without CD4. CD4+CCR5+ cells are the typical Th1cells that are prevalent at the sites of inflammation and this mechanism likely plays a pivotal role for recruitment and activation of T cells (24). After the cells attraction the CCR5 receptor is desensitized, possibly in order to terminate the process. Interestingly, the pre-treatment of cells with MIP-1β, SDF-1α/CXCR4 resulted in loss of responsiveness to IL-16 stimulation (23). In a diabetes mellitus (DM) model, T cells are attracted to the place of insulitis via CCL4, and inhibition of CCL4 and IL-16 were protective from DM (48).

### 2.2 IL-16 and B cells

Peripheral B cells express IL-16 at different proportions, ranging widely from 1.5%-94.4% (38). B cells express IL-16 mRNA and synthesize bioactive IL-16 protein, as observed in lymph node follicles (39). B cell supernatants induce migration of CD4+ Th cells, monocyte-derived DCs, and circulating DCs, which can be blocked via neutralization of IL-16. IL-16 seems important B-cell derived chemotactic factor involved in cellular cross-talk among T lymphocytes and DCs and their recruitment (39).

### 2.3 IL-16 and neutrophils

Neutrophils express IL-16 and are active secretors of IL-16, while IL-16 is a potent attractor of neutrophils to the site of inflammatory damage (40). In addition, secondary necrotic neutrophils may passively release interleukin-16 (41).

### 2.4 IL-16 in monocytes and macrophages

Human CD14+ monocytes constitutively express IL-16. If unstimulated within 6–8 h *in vitro* monocytes undergo apoptosis due to activation of casp-3, and release IL-16. It is not clear what is the stimulus and mechanism of this spontaneous release (42).

Monocyte cell line THP-1 infected with methicillin-resistant staphylococcus aureus (MRSA) secrete IL-16 (43), which can be blocked with anti-epidermal growth factor receptor (EGFR), anti-FasL antibodies (Ab), while pan-casp inhibitor (Z-VAD-FMK) or casp-3 inhibitors (43). In MRSA infected mouse lungs infiltrating immune cells express IL-16 and neutralizing anti-IL-16 Ab improved clearance of MRSA pneumonia in mice (43).

*In vitro*, stimulation with IFN-γ+LPS induce IL-16 mRNA upregulation in THP-1 monocyte cell line. Incubation of M0 macrophages with rIL-16 (150ng/ml) increase phagocytosis by 150%, and skew cytokine expression with upregulated IL-1α, IL-6 and IL-12, but downregulate IL-10; without affecting THP-1 cell proliferation (49).

### 2.5 IL-16 and dendritic cells and Langerhans cells

Interleukin-16 has an important role in DC differentiation. *In vitro* IL-16 can induce human cord blood cells and CD34(+) hematopoietic cells to proliferate and differentiate into phenotypically and functionally mature DCs (50). Monocyte exposure to IL-16 and thrombopoetin may lead to monocyte maturation to DCs (51).

In atopic dermatitis IgE-bound allergens are presented to epidermal LCs via the high affinity IgE receptor, FcϵRI and induces expression of IL-16, what results in chemoattraction of DCs, CD4+ T cells and eosinophils (44). Importantly casp-1, but not casp-3, further process IL-16 in epidermal DCs.

### 2.6 IL-16 and mast cells

Mast cells express CD9 and lack CD4 receptor. Ligation of CD9 by IL-16 on MCs induces their chemotaxis and activation (25). Cell treatment with anti-CD9 mAbs inhibit the IL-16–mediated chemotactic response of human (H) MC-1 line and its activation, same effects were observed on non-transformed human cord blood–derived MCs and mouse bone marrow–derived MCs by 50% to 60% (25).

### 2.7 IL-16 and natural killer cells

NK cells can also express and produce IL-16, and smokers display decreased levels of IL-16 in circulating NK cells. What biological function IL-16 mediates in NK cells requires further studies (38).

### 2.8 IL-16 and eosinophils

Interleukin-16 (IL-16) is known as a highly potent chemotactic and chemoattractant molecule for eosinophils (45). Tissue infiltrating eosinophils in eosinophilic conditions such as chronic eosinophilic rhinitis (ECRS) express IL-16 protein in parallel to other infiltrating cells such as MCs, lymphocytes and tissue epithelial cells. Data suggest that IL-16 may stimulate the migration and persistence of activated eosinophils in ECRS (46).

### 2.9 Epithelial cells

Keratinocytes and intestinal and respiratory epithelial and endothelial cells express IL-16 (52). The IL-16 ligand on these cells is CD9 receptor (15, 34, 35). What is the IL-16 function in or on these cells is not known.

### 2.10 Spontaneous release and targeted secretion of IL-16

Data indicate that IL-16 can be spontaneously released by unstimulated PBMCs in samples drawn and kept in rooms temperature. Importantly, this significant increase is observed in EDTA plasma, slighter increase in citrate plasma, but not at all in serum (53). Investigators suggested that abundant release of IL-16 in EDTA plasma could be due to presence of granulocytes as they are abundant in EDTA but not in citrate, and absent in serum. Investigators suggested that measuring IL-16 (and IL-8) in plasma could be an indicator of quality control if the samples were handled correctly prior to analysis. From the biological point of view these findings raise a relevant question: what environmental factor triggers release of IL-16 and if this process serves any physiologic function.

## 3 IL-16 in human disease

IL-16 has been implicated in multiple human conditions including inflammatory, autoimmune diseases, infections and cancers. Importantly, a recent major screening study for novel drug target evaluation identified IL-16 as a novel protein with causal evidence in multiple human diseases, a protein that has not been targeted so far (54). This finding sets a platform for this review, and IL-16 targeting could be of interest in multiple conditions. Overview data is summarized in **Table 2**.

**Table 2:** The expression patterns and studied functions of IL-16 in the different pathologies.

Condition	Levels in circulation*	Cell expression pattern	Functional associations and known impact	Ref.
AMI	↑ in ST-elevation	ND	Corr. with CRP, leukocytes, NT-proBNP, hs-troponin T	(55)
Heart failure	↑		Assoc. with myocardial fibrosis and stiffening	(55)
Rheumatoid arthritis	↑ synovial fluid and circulation	Synovial fluid and lining cells, fibroblasts	Attract CD4+ Levels increases in serum as indicator of response to treatment	(14, 56)
Psoriasis	↑	CD4 in skin inflammatory foci	Assoc with disease activity PASI	(57)
Inflammatory bowel disease	↑	Colon mucosa	Steroids treatment lower IL-16 in gut mucosa In murine colitis IL-16 inhibition reduce signs of gut inflammation	(58, 59)
Systemic lupus erythematosus	↑circulation and urine (in LN)	Inflammatory foci in the skin lesions and LN kidney	Corr. with disease activity	(60–62)
ANCA-vasculitis	↑		Assoc. with disease activity and damage	(63)
Takayashu vasculitis	↑	CD4+ cells in intramural infiltrates	Assoc. with active disease	(64)
Systemic sclerosis	↑	cells in skin infiltrates	Expression in skin assoc. with more severe disease	(65)
Idiopathic thrombocytopenic purpura	↑	ND	Levels decrease in serum after treatment	(66)
Grave’s disease	ND	Thyrocytes	ND	(67)
Diabetes mellitus	ND	ND	In murine model IL-16 mediate T cell attraction to insulitis	(48)
Cow milk protein allergy	↑	ND	Levels in serum decrease if allergen is avoided	(68)
Endometriosis	↑ endometric cysts		Assoc with pain	(69, 70)
Multiple sclerosis	↑	lesions CD4+, CD8+, CD83+, DCs and B cells	Levels in serum decrease after treatment Therapeutic effect of anti-IL-16 Ab in murine MS model	(71–74)
Depression Anxiety Insomnia Psychosomatic symptoms	↑		Assoc. with fatigue	(75, 76)
Pain		Murine models	Hyperalgesia, neuropathic pain	(77, 78)
Infections	↑	Range of cells	High levels biomarker of more severe disease Levels decrease at eradication Low levels or anti-IL-16 therapy assoc. with better outcomes	(79–85)
Malignancies		Loss of nuclear IL-16 function Extracellular Il-16	Loss of cell cycle control Attracts but suppresses antitumor immunity	(86–92)

* - if not stated otherwise. ↑ - increased levels. Ref., reference; AMI, Acute myocardial infarction; assoc., associate; corr., correlate; CRP, C-reactive protein; NT-proBNP, N-terminal pro–B-type natriuretic peptide; hs-troponin T high sensitivity troponin T; PASI, psoriasis activity and severity index; LN, lupus nephritis; ANCA, antineutrophil cytoplasmic antibody; MS, multiple sclerosis; Ab, antibody.

### 3.1 Acute myocardial infarction and heart failure

Increased circulating levels of IL-16 are found in patients with ST-elevation AMI, with those without ST-elevation having lower circulating levels (55). In addition, IL-16 levels correlated positively with C-reactive protein (CRP), leukocyte count, NT-proBNP, high sensitivity troponin T levels and negatively with amount of circulating DCs (55). Interleukin-16 (IL-16) has also been reported to mediate left ventricular myocardial fibrosis and stiffening in patients with heart failure with preserved ejection fraction (55).

### 3.2 Rheumatoid arthritis

The importance of IL-16 in rheumatoid arthritis (RA) has been demonstrated by several investigators (93, 94). High levels of IL-16 are found in the synovial fluid of RA joints and the lining layer of RA synovia (27). It is considered that IL-16 is produced by fibroblasts and attracts CD4+ cells to the synovial infiltrates (14). *In vitro*, dexamethasone treatment blocked RA patient derived immunoglobulins (IgG) induced T cell migration and IL-16 expression (94). Importantly in SOMAscan™ assay IL-16 was identified out of 1128 serum proteins as the best predictor of treatment response at 12 weeks: IL-16 levels were significantly reduced after MTX treatment in MTX-naïve patients, and by abatacept or tocilizumab treatment in patients with inadequate response to MTX (56).

### 3.3 Psoriasis

In psoriasis, a common immune-mediated skin disease, serum levels of IL-16 correlate with disease severity measured by psoriasis area and severity index (PASI). Cutaneous inflammatory infiltrates have abundant IL-16 carrying and producing cells, the majority of which express CD4 (57).

### 3.4 Inflammatory bowel disease

Increased levels of IL-16 are observed in colonic mucosal biopsies in patients with Crohn disease and ulcerative colitis. Treatment with steroids efficiently reduced IL-16 expression in the gut mucosa to the levels as low as in controls (58). Anti–IL-16 mAb treatment significantly reduced signs of disease in an animal colitis model (trinitrobenzenensulfonic acid (TNBS)-induced), such as weight loss, mucosal ulceration, myeloperoxidase activity (*P* < 0.001), as well as reduced mucosal levels of IL-1β and tumor necrosis factor-α (TNF-α) (59).

### 3.5 Systemic lupus erythematosus

Implications of the IL-16 role in SLE were reported several decades ago (95). Lately, two independent groups identified IL-16 as the key cytokine in cutaneous and renal involvement in lupus erythematosus (60–62), and also in mouse model of SLE-like pulmonary inflammation (40). Our group has performed an unbiased proteome analysis of cutaneous inflammatory infiltrates in cutaneous LE (CLE), and IL-16 was identified as the top expressed cytokine (96). A urine proteome study using unbiased methods identified IL-16 as a marker of the most severe lupus nephritis (62). We independently confirmed their findings using ELISA and immunohistochemistry in Swedish SLE cohort (60). Independent investigators suggested IL-16 as pro-inflammatory driver, being a SLE disease biomarker of severe renal engagement, and have proposed it as a promising therapeutic target (60–62).

### 3.6 Vasculitis

Antineutrophil cytoplasmic antibody associated (ANCA) vasculitis, a group of rare, but life threatening, systemic diseases affecting small vessels, including those of vital organs, has been associated with high levels of circulating IL-16; in addition, circulating IL-16 levels correlated with disease damage (63). In Takayashu vasculitis, inflammation affects the walls of large vessels and IL-16 expression was observed both in the circulation and intramural inflammatory infiltrates, and IL-16 levels correlated with amounts of CD4+ cells infiltrating large vessel walls, which induce arterial lesions (64).

### 3.7 Systemic sclerosis

SSc (also known as scleroderma), a rheumatic disease manifesting as vasculopathy and fibrosis of connective tissue. The serum IL-16 levels in SSc patients are significantly elevated (65). In the diffuse form, the amount of skin infiltrating IL-16 positive cells were higher than in those with the limited SSc form or controls, indicating that high IL-16 associate with a more severe SSc (65).

### 3.8 Idiopathic thrombocytopenic purpura

In patients with ITP autoimmune mechanisms destroy thrombocytes and confer risk for major bleeding, while purpura is an early diagnostic sign. Increased levels of IL-16 are observed in circulation of patients with ITP, and levels decrease after high dose dexamethasone therapy (66). Besides high protein concentrations, IL-16 mRNA was also found to be upregulated and to correspond to casp-3 mRNA levels, which also dropped after treatment (66).

### 3.9 Graves’ disease

In GD autoimmune inflammation target the thyroid and connective tissues in the orbit where auto-Abs induce hyperfunction, inflammation and subsequent risk of destruction and hypofunction of thyroid gland. Thyrocytes express mRNA for IL-16, and casp-3 and proIL-16 can be detected in thyrocyte cultures (67). Immunoglobulin (Ig)G from patients with GD induces IL-16 and the chemokine RANTES expression in cultured human thyrocytes, which may contribute to lymphocyte recruitment to the thyroid and promotion of autoimmune insult (97).

### 3.10 Cow milk protein allergy

Infants who develop CMPA have cytokine disbalance: increased IL-16, and IL-17A, decreased IL-12, while cow milk avoidance led to increase of IL-12, and decrease in IL-16 and IL-17A. Serum IL-12, IL-16, and IL-17A levels have been proposed as diagnostic biomarkers in CMPA (68).

### 3.11 Endometriosis

Endometriosis, a gynecological condition where ectopic endometrial foci induce inflammation, abdominal pain and reduced fertility, is associated with high peritoneal IL-16 (69). In an unbiased analysis of endometriotic cyst fluid proteins, IL-16 ranked as third most abundant cytokine. Moreover investigators discovered that IL-16 release in endometrial cysts is a result of iron overload induced cell pyroptosis (70) (**Figure 2**). Abundant IL-16 contributes to local inflammation and abdominal pain in endometriosis.

As reviewed above, circulating levels of IL-16 are increased in multiple autoimmune diseases, however studies demonstrated impaired or defected function of casp-3 or regulation or expression of IL-16 in several autoimmune conditions, what could subsequently lead to defective IL-16 processing and secretion (98). The hypothesis, that there could be alternative mechanisms of IL-16 release is supported by findings of the recent study in endometriosis as pyroptosis could be one of novel pathways of IL-16 secretion (70). Interestingly, pyroptosis is implicated in multiple autoimmune diseases, as triggers that activate inflammasome may lead to pyroptosis.

### 3.12 Neuronal IL-16 and its role in inflammatory diseases of nervous system, psychiatric conditions and pain

A larger splice variant (141 kD) of IL-16, found within CD4+ post-mitotic granule neurons in the cerebellum and hippocampus, is entitled neuronal IL-16 (NIL-16) (21). Similarly to pro-IL-16, proN-IL-16 is posttranslationally cleaved by casp-3. The NIL-16 contains two additional PDZ domains in its N-terminal portion, which interact with neuronal ion channels, while the C-terminal portion is identical to the lymphocyte bioactive IL-16. The role of NIL-16 in maintenance of the processes in CNS has been explored in several studies in human conditions (71).

#### 3.12.1 Multiple sclerosis

MS, a chronic inflammatory demyelinating and degenerative disease of the CNS, also appears to be affected by IL-16 (71). In the brain lesions of relapsing and remitting (RRMS) lesions CD4+, CD8+, CD83+, DCs and B cells have been observed to express IL-16, as well as casp-3 (71). Circulating levels of IL-16 correlate to markers of axonal damage (such as medium and heavy chains of neurofilaments) and cells carrying IL-16 are found in proximity to MS lesions (72), while levels of serum IL-16 drop after successful treatment with IFNβ-1a and was suggested as a marker of clinical response (73). Anti-IL-16 Ab injection had therapeutic value in a mouse model of MS (EAE) (74).

#### 3.12.2 Psychiatric conditions

Depressive symptoms and major depressive disorder, anxiety, insomnia and phsychosomatic symptoms, fatigue-fibromyalgia symptoms and melancholia have been found to be associated with circulating levels of IL-16 and activation of Th1 immune response (75, 76). Authors suggested that IL-16 could be seen as “psychocutaneous” cytokine and could link depression in psoriasis patients. Based to the finding of the current study on association of IL-16 and fatigue-fibromyalgia symptoms, one could hypothesize that high IL-16 levels could stand for fatigue symptoms common in patients with other autoimmune, inflammatory diseases or even cancer, and coins further studies.

#### 3.12.3 IL-16 and pain

Several investigators studied the role of IL-16 in mouse nociception. Both local and systemic administration of IL-16 in mouse induce hyperalgesia, which can be inhibited by non-steroid anti-inflammatory drugs or IL-16 neutralizing Ab (77). In another mouse model of nociceptive pain, IL-16 has been identified to be involved in astrocyte and glial activation and enhanced nociceptive neuron excitability and promoted inflammatory pain (78). In a mouse model of neuropathic pain induced by using spinal nerve ligation, it was found female specific IL-16 mRNA upregulation (29). In this mouse model, stimulation with IL-16 induced astrocyte activation via CD4+ T cells and targeting this pathway was proposed as a therapeutic possibility for future treatment of neuropathic pain in females (29).

### 3.13 IL-16 in severe human infections

Increased levels of IL-16 are found in multiple infections. In *Clostridium difficile* infection it was suggested as a biomarker of more severe disease (79). Cells overexpressing IL-16 are resistant to HIV-1 infection, and IL-16 has been explored as a co-enhancer of HIV-1 virus eradication (80, 81). Low expression of IL-16, among other cytokines, was one of several identified factors for poor outcomes of COVID-19 (82). IL-16 seems to have a specific role in *Mycobacterium tuberculosis* (Mtb) infection, since IL-16 is produced by Mtb infected macrophages, but may inhibit phagosome maturation and uptake of Mtb and promote the intracellular survival of the pathogen. The recent study found that Mtb hijacks the host-macrophages derived IL-16 and suggested that IL-16 might have immunosuppressive role in Mtb infection (83). In sepsis, circulating levels of IL-16 are increased, while neutralization of IL-16 with Abs reduced mouse mortality and sepsis induced cardiac injury (84). Different single nucleotide polymorphisms of IL-16 are associated with susceptibility for hepatitis B and C infections (5, 85).

### 3.14 IL-16 in human malignancies

The issue of IL-16 and malignancy, first reviewed in 2014 (34), has since been the topic of over 100 original papers. In brief, a role of IL-16 has been identified in multiple malignancies, including hematological and solid tumors. The role of IL-16 in tumors could be mediated by different functions or loss thereof.

The importance of IL-16 in regulation of cell cycle and growth is underscored by the findings that the cells of certain cancers lack the IL-16 gene or have mutations in nuclear localization sequence of the protein, preventing its entrance to the nucleus and resulting in hyperproliferative states (86, 87), as was demonstrated in cutaneous T cell lymphoma (CTCL) (88). In mycosis fungoides IL-16 was demonstrated to attract malignant effector memory T cells (CCR4+TSLPR+CD4+CCR7-CD31+) to the skin (89).

Loss of nuclear IL-16 in cancer cells has been associated with increase in cytoplasmic IL-16, which has been considered to act as a growth factor for malignant T cells, and also was associated with decreased rate of cancer transformed cell apoptosis (34). Overexpression of IL-16 in multiple myeloma bone marrow has been showed to function as growth factor for CD4 and C9 expressing malignant plasma cells (90). Addition of neutralizing anti-IL-16 Abs resulted in significant reduction of cell proliferation.

In bladder cancer it was shown that IL-16 enhances the suppressive T regs capacity within sentinel lymph nodes and could contribute as tumor immune escape mechanism (91). Extracellular IL-16 in cancer patients is a potent chemoattractant of immune cells and that IL-16 participates in development of immunotolerance to malignant cells, since inhibition of IL-16 improved prognosis in those models (92). Blocking or neutralizing IL-16 could enhance antitumor effects of several substances and has been proposed as an adjuvant target by several investigators (99). For example tumor intrinsic Aurora-A promotes the cytotoxic activity of CD8+T cells in immune-hot chemotherapy resistant cancer (CRC) via negative regulation of interleukin-16 (IL-16). The upregulation of IL-16 was suggested as tumor escape mechanism and impaired the therapeutic effect of Aurora-A inhibition. Neutralization of IL-16 could promote therapeutic effect of Aurora-A inhibitor (99).

## 4 IL-16 and pyroptosis

As reviewed above, circulating levels of IL-16 are increased in multiple autoimmune diseases, which have also been associated with impaired expression and regulation of both casp-3 and IL-16 (98). Thus, there could be alternative mechanisms of IL-16 cleavage and release. A recent study in endometriosis demonstrated that IL-16 can be cleaved and released during iron overload induced pyroptotic cell death via non-classical GASDME mediated pathway (70). Interestingly, pyroptosis is implicated in multiple autoimmune diseases and common triggers of autoimmunity such as immune-complexes, secondary necrotic cell debris, extracellular damaged DNA could possibly trigger pyroptosis, and hypothetical IL-16 release (**Figure 2**) (100).

In cancer, the expression of GASDME is lower in most tumor cells due to the epigenetic inactivation caused by methylation, while chemotherapy may activate casp-3, GADSME expression and facilitate tumor cell pyroptosis (101, 102). It is not clear if cancer pyroptotic cell death results in release of cleaved IL-16, as was proven in the case in endometriosis (**Figure 2**) (70).

## 5 Genetic polymorphisms of IL-16 gene

Several polymorphisms of IL-16 gene have been associated with cancer and autoimmune diseases. Two meta-analysis confirmed association of cancers with rs11556218T>G, and one meta-analysis confirmed association of cancers with rs4778889T>C polymorphisms. The rs11556218T>G polymorphism was also associated with the risk of cardiovascular disease in Chinese population (103, 104). Genetic studies in autoimmune diseases found associations with polymorphisms in the same loci, but did not reach genome-wide association study power and will not be reviewed in this paper.

## 6 Successful outcomes in IL-16 inhibition in mouse models of human diseases

So far inhibition of IL-16 has been addressed only in animal models. In a model of spontaneous diabetes in NOD female mice, blockade of IL-16 with neutralizing Abs prevented insulitis, destruction of insulin-producing pancreatic islets and inhibited development of type 1 diabetes (48). Experimental autoimmune encephalomyelitis (EAE) is the typical model to study multiple sclerosis. In this model the majority of the immune cells infiltrating central nervous system express IL-16. Blockade of IL-16 with neutralizing Ab led to successful outcomes - reversed paralysis, ameliorated relapsing disease, reduced infiltration by CD4+ T cells and demyelination (74). In models of sepsis or cardiac injury, blockade of IL-16 reduced mortality and improved survival and reduced damage via activation of antioxidant pathways (84, 105). Importantly neutralization of IL-16 proved adjuvant effect on novel cytostatic drugs and could improve outcomes in oncologic diseases (99).

## 7 Possible therapeutic approaches to IL-16 inhibition

**Figure 3:**
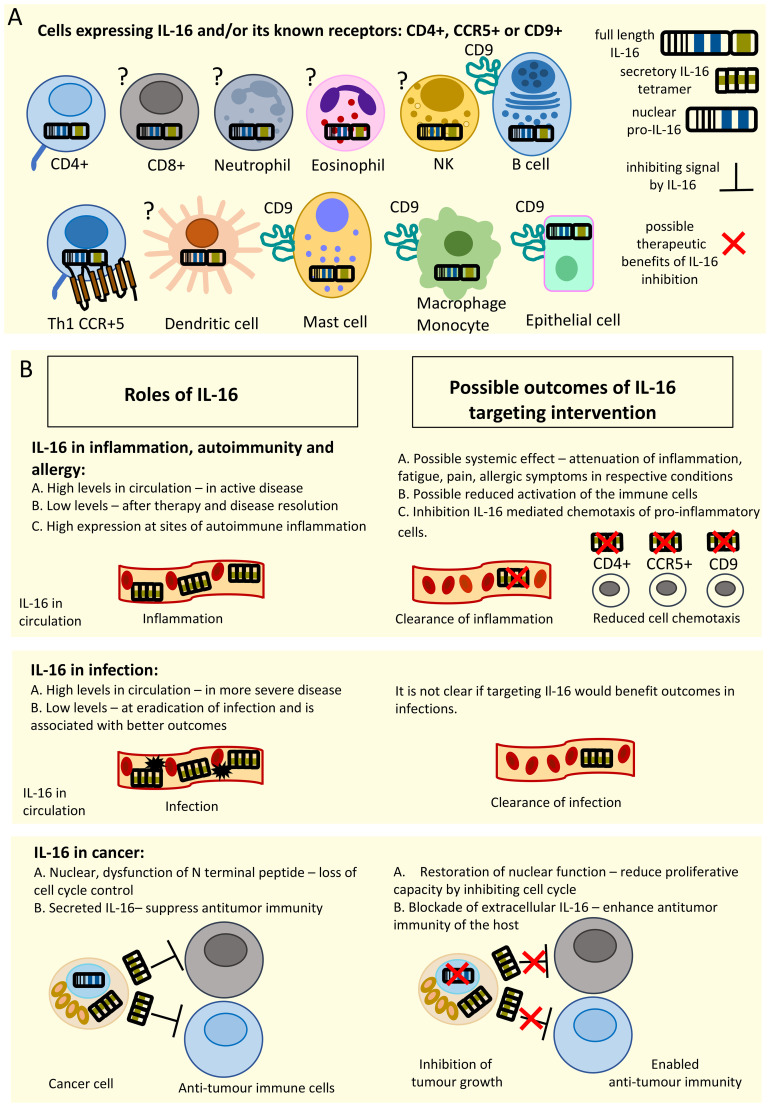
Graphical abstract. Panel **(A)** illustrates what immune cells express IL-16 and its receptors. ? – indicates that it is not clear if the cell type expresses any receptor for IL-16. NK - natural killer cell, Th1 – T helper 1 cell. Panel **(B)** illustrates overview of IL-16 roles in inflammation, infection and cancer (left) and possible outcomes of therapeutic IL-16 inhibition (right): neutralization of extracellular IL-16 could attenuate inflammation and cell recruitment to the inflammatory sites (upper panel); there is no clear information whether IL-16 targeting could benefit infections (middle panel); in cancers IL-16 inhibition could allow anti-tumour immune response and possibly block cell cycle progression (lower panel).

Neutralization of cytokines with *in vitro* produced anti-cytokine Abs has been used in daily practice in rheumatology and cancer care over the last several decades. It could be one of the modalities chosen for therapeutic inhibition of IL-16 function, as in experimental animal models described in section 6 anti-IL16 neutralizing Abs showed positive results (48, 74, 84, 99, 105). An alternative approach could be to block IL-16 receptor with anti-receptor Abs. The inhibition of CD4 receptor is not possible, as this receptor is vital for its role in protection against infections. However inhibition of CD9 or CD4 co-receptor CCR5 could be novel potential target. Hypothetically inhibition of CCR5 could reduce the potency of IL-16 stimulus to CD4 and could still guarantee the vital IL-16 signal. Data on hypothesis of possible therapeutic interventions is summarized in graphical abstract (**Figure 3**).

In conclusion, the IL-16 protein has multiple functions in the human immune system, and impaired protein regulation or function or genetic polymorphisms are associated with a variety of life-threatening acute and chronic conditions. Overall data in inflammatory diseases indicate that inhibition of IL-16 could attenuate inflammatory responses and reduce recruitment of target cells to the inflammatory sites. In cancers, loss of nuclear IL-16 leads to hyperproliferative states, which are more difficult to target, but neutralization of secreted IL-16 could improve outcomes of chemotherapy and modulate tumor escape mechanism.
